# Extracellular vesicles in COVID-19 convalescence can regulate T cell metabolism and function

**DOI:** 10.1016/j.isci.2023.107280

**Published:** 2023-07-04

**Authors:** Molly S. George, Jenifer Sanchez, Christina Rollings, David Fear, Peter Irving, Linda V. Sinclair, Anna Schurich

**Affiliations:** 1 Department of Infectious Diseases, School of Immunology and Microbial Sciences, King’s College London, London SE1 9RT, UK; 2 Cell Signalling and Immunology, School of Life Sciences, University of Dundee, Scotland DD1 5EH, UK; 3 Peter Gorer Department of Immunobiology, School of Immunology and Microbial Sciences, King’s College London, London SE1 9RT, UK; 4 Department of Gastroenterology, Guy’s and St Thomas’ Hospital, London SE1 9RT, UK

**Keywords:** Immunology, Virology

## Abstract

Long-term T cell dysregulation has been reported following COVID-19 disease. Prolonged T cell activation is associated with disease severity and may be implicated in producing long-covid symptoms. Here, we assess the role of extracellular vesicles (EV) in regulating T cell function over several weeks post COVID-19 disease. We find that alterations in cellular origin and protein content of EV in COVID-19 convalescence are linked to initial disease severity. We demonstrate that convalescent donor-derived EV can alter the function and metabolic rewiring of CD4 and CD8 T cells. Of note, EV following mild, but not severe disease, show distinctly immune-suppressive properties, reducing T cell effector cytokine production and glucose metabolism. Mechanistically our data indicate the involvement of EV-surface ICAM-1 in facilitating EV—T cell interaction. Our data demonstrate that circulatory EV are phenotypically and functionally altered several weeks following acute infection, suggesting a role for EV as long-term immune modulators.

## Highlights

• EV profiles remain distinct at several weeks following COVID-19 disease
• Co-culture with EV impacts healthy T cell effector-functions and metabolism *in vitro*
• EV from patients post mild COVID-19 disease have suppressive capacity
• EV can interact with T cells via ICAM-1/LFA-1 binding

## Graphical abstract

**Figure:**
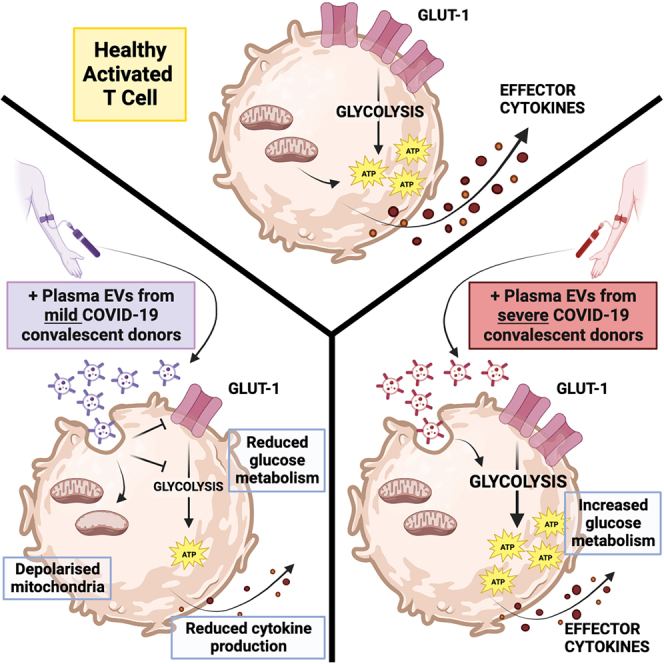

## Introduction

Severe acute respiratory syndrome coronavirus 2 (SARS-CoV-2) has caused more than 600 million infections, 6 million deaths and left an unknown number of individuals suffering from long-COVID-19 sequelae. T cells are involved in acute viral clearance and protection against future infections.^1^ Recent studies demonstrate that T cell phenotype and functions remained altered post SARS-CoV-2 infection, with alterations associated with initial disease severity and prevalence of long-term symptoms. Robust T cell immunity is induced in convalescent individuals following mild COVID-19 disease,^2^ yet in convalescence following severe COVID-19 the T cell response is found to be dysregulated. Several publications report excessive proinflammatory T cell cytokine production at 3–6 months post-infection^3^^,^^4^^,^^5^ and long-term expression of T cell exhaustion markers.^6^^,^^7^ How these T cell alterations are regulated and the role of cell-cell communication in COVID-19 convalescence is not well understood.

Extracellular vesicles (EV) have received increased interest in recent years as important mediators of intercellular communication.^8^^,^^9^ EV have been detected in all body fluids and carry a range of cargo shown to impact recipient cells, including lipids, metabolites, nucleic acids and various proteins, and enzymes.^8^ It has recently been shown that the composition of EV cargo proteins is cell-type and tissue specific.^10^ The quantity of EV produced by cells is further influenced by cellular environment and activation status.^10^ Multiple studies have explored the impact of EV on immune cells during disease. In line with a context-dependent communication, both immune suppressive and immune-activating functions have been proposed, for example, suppression of T cell function mediated by cancer-derived EV^9^^,^^11^^,^^12^ and stimulation of T cells by dendritic cell-derived EV.^13^ Multiple roles for EV have also been described during active SARS-CoV-2 infection.^14^^,^^15^^,^^16^^,^^17^^,^^18^^,^^19^^,^^20^ However, the potential role of EV in modulating the immune system in COVID-19 convalescence remains to be assessed.

Here, we assess the cellular origin of circulating EV in convalescent individuals, post mild or severe COVID-19 disease and as controls in healthy unexposed and healthy vaccinated donors. We investigate the capacity of these EV to regulate healthy human T cell function post-COVID-19. Our data reveal distinct compositions of EV, loaded with different protein cargo, in the individual donor groups. EV differentially impacted on T cell activation and metabolic programming, resulting in altered regulation of T cell effector function. EV derived from donors post-mild COVID-19 disease confer a uniquely suppressive capacity. Our findings demonstrate that EV have the capacity to influence the immune response for a prolonged period post immune activation.

## Results

### Circulatory EV carry distinct surface markers in COVID-19 convalescent and vaccinated donors

First, we sought to determine the composition of EV in circulation several weeks following SARS-CoV2 infection. Non-exposed healthy individuals (to the donor’s knowledge) were recruited as controls at the start of the pandemic. However, with the introduction of COVID-19 vaccinations, it soon became apparent that vaccination impacted the results. Therefore, we obtained historic (Biobanked pre-2019) healthy control samples and specifically recruited individuals post-vaccination as an additional control group. Briefly, EV were purified from the plasma of individuals 4–10 weeks after recovery from either mild COVID-19 disease (Mild EV), severe COVID-19 disease requiring hospitalization (Severe EV) or 2–10 weeks following Pfizer/BioNTech SARS-CoV2 mRNA COMIRNATY vaccination (Vac EV) or from healthy unexposed/unvaccinated donors (HD EV) (donor data in Table 1, STAR methods). To avoid co-isolating small non-EV particles during ultra-centrifugation, we instead isolated plasma EV by immune-bead separation targeting the tetraspanin CD63; a protein commonly found on EV.^21^ To evaluate the composition and cellular origin of plasma-derived EV, we attributed the “parent” cell derivation of the EV by the expression of key determining proteins (e.g., CD4/CD8 for T cells, CD19 for B cells, Figure S1A).

**Table 1:** Demographics and clinical information of recruited donors (∗median and range shown)

Donor	No.	Sex (% Male)	Age∗	Days since symptoms or vaccination∗	Vaccine given	COVID test	COVID symptoms	Material Used
Healthy Donor (HD)	13	40	50 (26–63)	N/A	none	none	none	Plasma and PBMCs
Vaccinated (Vac)	8	50	37 (25–60)	41 (12–71)	COMIR-NATY	none	none	Plasma only
Mild COVID-19 convalescent (Mild)	14	14	47 (28–62)	54 (30–71)	none	pos	None - mild cough/aches	Plasma only
Severe COVID-19 convalescent (Severe)	12	36	53 (43–77)	50 (34–77)	none	pos	Hospitalized with fever/breathing difficulties	Plasma only

**Figure 1:**
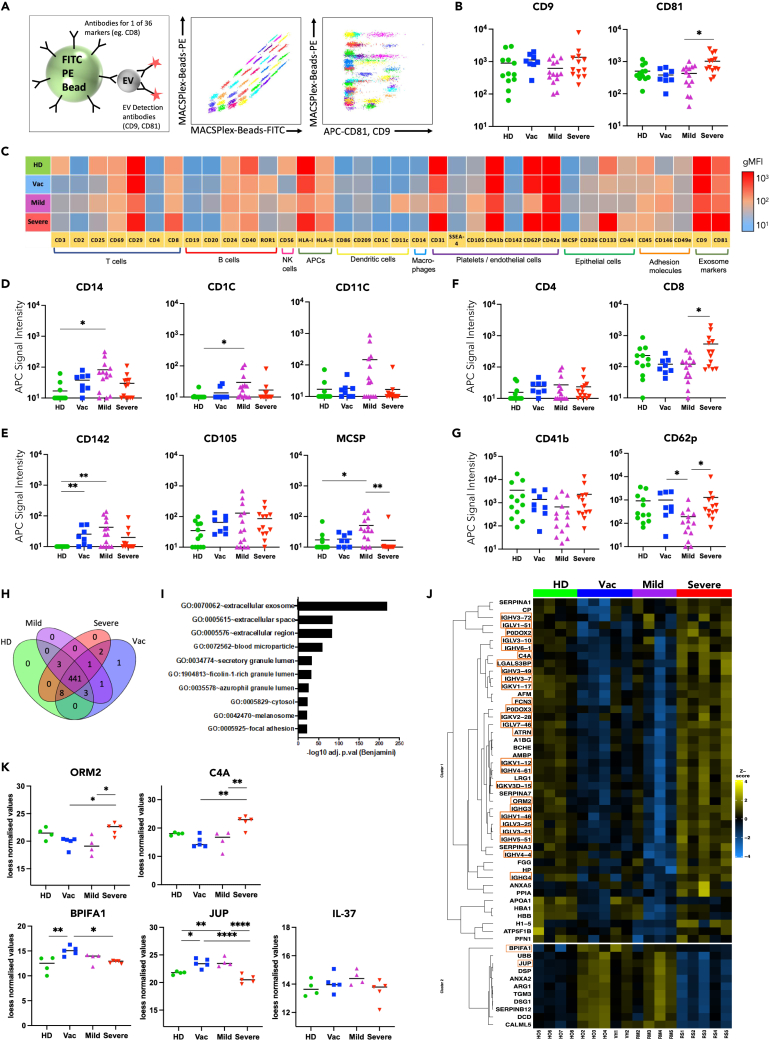
Plasma-derived EVs carry distinct surface and intra-vesicle proteins in COVID-19 convalescent donors (A) Outline of EV surface marker detection by multiplexed flow cytometry (MACSPlex) with plot showing the 36 antibody-coated beads + 3 control beads distinguishable by PE and FITC fluorescence and EV detection by CD9-APC and CD81-APC. (B) Geometric mean fluorescence intensity signal (GMFI) for EV markers CD9 and CD81. (C) Overview of the GMFI for 36 surface markers on EV derived from donors that are healthy (HD, n = 13), vaccinated (Vac, n = 8), recovered from mild COVID-19 disease (Mild, n = 9) or recovered from severe COVID-19 disease (Severe, n = 12). Summary data for surface markers analyzed by MACSPlex. (D) CD14, CD1C, CD11C (E) CD142, CD105, MCSP (F) CD4 and CD8 (G) CD41b, CD62p. One-way ANOVA used to compare EV from different donor groups ∗p < 0.05, ∗∗p < 0.01. (H) Number of proteins identified by mass spectrometry analysis of intra-vesicle cargo in EV derived from HD (n = 4), Vac (n = 5), Mild (n = 4) and Severe (n = 5) donors. (I) Gene-ontology cellular component analysis of total identified proteins across all groups. (J) Heatmap of differentially expressed intra-vesicle proteins calculated from loess normalized values, orange boxes signify proteins with known immune-modulatory function. (K) Loess normalized expression values for selected proteins. Groups compared by one-way ANOVA ∗p < 0.05, ∗∗p < 0.01, ∗∗∗∗p < 0.0001.

We used multiplex flow cytometry (MACSPlex) to assess 36 individual standard cell surface markers simultaneously (Figure 1A). All samples had a clear EV signature, with similar levels of tetraspanins CD9 and CD81 detectable (Figure 1B), with the exception of EV derived from severe recovered donors that had a higher abundance of CD81^+^ EV as compared to other donor samples (Figure 1B). Several weeks after initial immune activation, EV surface antigens differed notably between the groups (Figure 1C). In healthy donors, we detected CD8^+^ EV, as well as platelet and endothelial cell-derived EV (CD62p^+^, CD41b^+^, CD42a^+^) whereas other markers such as CD4 were weakly expressed (Figures 1C–1G). EV derived from mild recovered donors were characterized by a high abundance of antigen presenting cell-derived markers (CD14^+^, CD1C^+^, CD11C^+^) (Figure 1D) and a high proportion of endothelial and epithelial cell-derived markers (CD142^+^, CD105^+^, MCSP^+^) (Figure 1E). In contrast, Severe EV were characterized by a high abundance of both T cell-derived markers (cytotoxic CD8^+^ as well as helper CD4^+^) and platelet-derived markers (CD41b^+^, CD62p^+^) (Figures 1F and 1G). EV derived from vaccinated donors did not fully align with either of these phenotypes, being abundant in endothelial cell-derived markers (CD142^+^, CD105^+^) akin to Mild EV (Figure 1E), but also sharing similarity with Severe EV, characterized by a high abundance of the helper T cell-derived marker CD4 (Figure 1F), as well as presenting additional markers CD29^+^, HLA-class I^+^, and CD31^+^ (Figure 1C). Our data thus suggest that following immune activation by vaccination, mild or severe infection or at assumed homeostasis in healthy controls, distinct sets of cell types predominantly produce circulating EV in each group.

Indeed, following mild or severe COVID-19 disease donors could be clustered based on their EV surface marker composition (Figures S1B and S1C). EV surface marker profile of Mild and Severe EV in our cohort did not cluster by donor age or sex (Figure S1C).

### Mild and severe COVID-19 convalescent EV contain differential protein cargo

Having observed clear differences in the expression of surface proteins on COVID-19 convalescent EV, we next assessed whether intra-vesicle protein abundances were also altered. To this end, we investigated EV protein cargo using a proteomics protocol to specifically and sensitively profile intra-EV proteins. We were able to detect 460 proteins across our samples, with high overlap in detectable proteins between HD, Vac, Mild, and Severe EV (Figure 1H). Intra-EV proteomics confirmed a strong EV signature by Gene Ontology Cellular Component analysis, with “extracellular exosome” being the top hit across all samples (Figure 1I). We compared protein abundances in HD, Vac, Mild, and Severe donors by hierarchical clustering (Figure 1J) and found that, in line with EV surface marker profiling, EV from mild and severe COVID-19 convalescent donors were the most different. Interestingly, Vac EV shared similarity with Mild EV in terms of protein content. We found many of the differentially expressed proteins to have immune-modulatory functions (Figure 1J), with some of those that are highly packaged in Severe donor EV having roles in innate immune activation. This includes a number of immunoglobulins (Figure 1J) as well as other acute phase proteins such as complement component 4 (C4A) and orosomucoid-2 (ORM2) (Figure 1K), of which high levels have been correlated with severe COVID-19 disease.^22^ In contrast, the immune-modulatory proteins that were more highly packaged in Vac and Mild EV have reported roles in immune suppression, such as BPI Fold Containing Family A Member 1 (BPIFA1) which can suppress epithelial cell inflammatory cytokine production^23^ and γ-catenin (junction plakoglobin, JUP) (Figure 1K) which is a suppressor of the WNT signaling pathway and inducer of anti-inflammatory cytokine IFN-β.^24^ Additionally, we found interleukin-37 (IL-37) to be present, with a trend of being increased in mild and decreased in severe EV (Figure 1K). IL-37 is elevated in regulatory T cells^25^ and can suppress T cell cytokine production through intracellular binding to pSMAD3.^26^ Overall, there was a trend toward EV carrying pro-inflammatory proteins in severe COVID-19 convalescence versus immune-suppressive proteins in mild COVID-19 convalescence.

### EV from mild COVID-19 convalescent donors suppress T cell effector function

**Figure 2:**
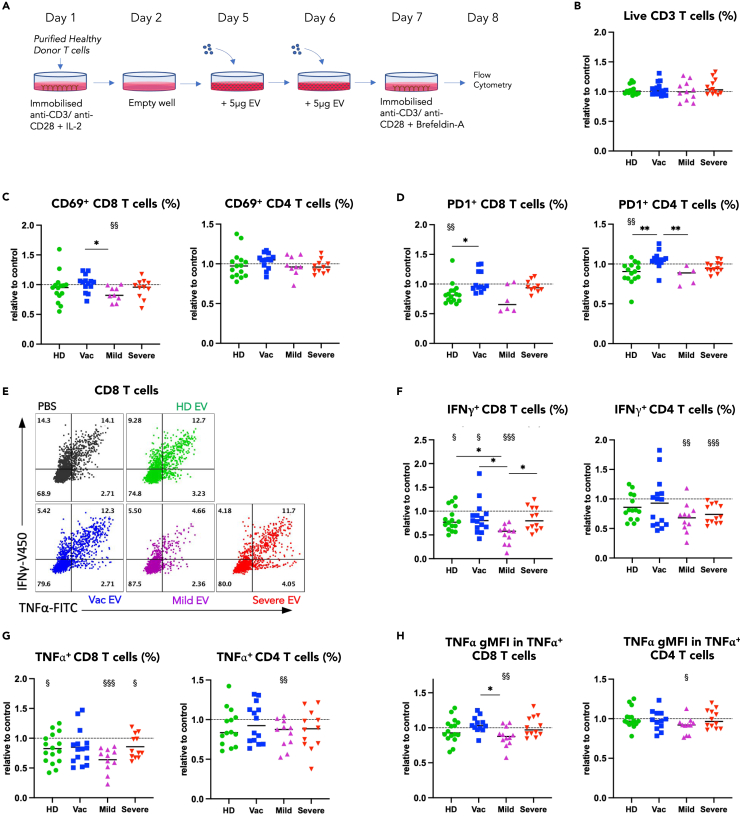
EV derived from COVID-19 convalescent donors differentially impact the effector function of healthy CD8 and CD4 T cells (A) T cells were isolated and stimulated overnight with plate bound anti-CD3 and anti-CD28, on day 2 T cells were removed from activation, 5 μg EV derived from the different donors were added on day 5 and 6, T cells were restimulated prior to analysis by flow cytometry. All flow cytometry data are shown as values relative to the PBS control within the same experiment. (B) Relative proportion of live T cells following EV incubation as specified. (C) Relative proportion of CD69^+^ and (D) proportion of PD1^+^ CD8 an CD4 T cells following EV incubation. (E) Representative example of flow cytometric analysis of IFNγ^+^ and TNFα^+^ CD8 T cells following incubation with EV as specified, with summary data for relative proportion of (F) IFNγ^+^ (G) TNFα^+^ and (H) Relative GMFI TNFα in TNFα^+^ CD8 and CD4 T cells. One-way ANOVA used to compare effect of EV from different donor groups ∗p < 0.05, ∗∗p < 0.01. Wilcoxon signed rank test used to compare groups with the PBS control (i.e., to 1) §p < 0.05, §§p < 0.01, §§§p < 0.001.

Following the intriguing finding that circulatory EV were different in composition at several weeks post COVID-19 disease, we were interested to understand whether EV could influence immune cell responses. T cell responses have been shown to be distinctly altered in mild versus severe COVID-19 disease and have also been implicated in symptoms of long-COVID.^27^ Based on previous protocols to study human T cell function in culture,^28^^,^^29^ we established an *in vitro* method to co-culture EV with activated healthy donor-derived T cells to assess the impact of EV on T cell function (Figure 2A). Healthy donor T cells were used for co-culture with the reasoning that these were not already impacted by “immune EV modulation” *in vivo*. All experiments were normalized to responses of healthy donor T cells treated with vehicle (PBS control).

Addition of EV did not impact T cell viability during culture (Figure 2B). First, we assessed the impact of EV on T cell activation in culture as measured by T cell expression of the early activation marker CD69 and activation/inhibitory receptor programmed death-1 (PD-1). EV derived from vaccinated donors showed a trend of enhancing healthy donor T cell activation, while Mild EV tended to decrease the proportion of both CD69^+^ and PD-1^+^ T cells (Figures 2C and 2D) relative to the PBS control. Next, we investigated the impact of EV on T cell anti-viral functions as measured by production of the effector cytokines IFNγ and TNFα. We found that EV derived from mild convalescent donors significantly decreased the frequency of IFNγ- and TNFα-producing CD8 T cells and CD4 T cells (Figures 2E–2G). Mild EV also negatively impacted the production of TNFα on a per cell basis in both CD8 and CD4 T cells as measured by a decrease in geometric mean fluorescence intensity (gMFI) (Figure 2H). Vac EV induced a weaker but significant decrease in the frequency of IFNγ- and TNFα-producing CD8 T cells but were unable to induce suppression of CD4 T cells. In contrast, EV from HD and Severe-recovered donors mostly did not alter T cell activation and cytokine production.

### EV can alter the metabolic program of healthy T cells

**Figure 3:**
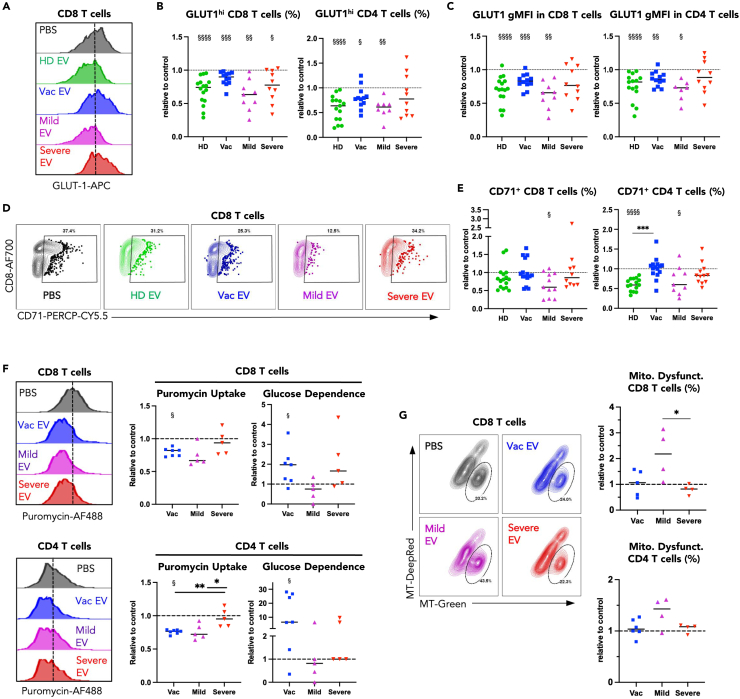
EV derived from COVID-19 convalescent donors differentially impact the metabolism of healthy CD8 and CD4 T cells T cells stimulated and treated with EV as in Figure 4A then assessed by flow cytometry. All flow cytometry data are shown as values relative to the PBS control within the same experiment. (A) Representative example of flow cytometric analysis of GLUT-1 in CD8 T cells following incubation with specified EV, with summary data for (B) relative GMFI GLUT-1 and relative proportion of GLUT-1^hi^ CD8 and (C) CD4 T cells. (D) Representative example of flow cytometric analysis of CD71^+^ CD8 T cells following incubation with EV as specified, with summary data for (E) relative proportion of CD71^+^ CD8 and CD4 T cells. (F) CD8 and CD4 T cell functional metabolism was assessed by incubation with puromycin which is incorporated into newly synthesized peptides. Glucose dependence was determined by suppression of puromycin uptake induced upon 2-DG incubation—further details in the STAR methods section and in Figure S2. (G) Representative example of flow cytometric analysis of dysfunctional mitochondria^+^ CD8 T cells by staining with mitotracker-red and -green following incubation with EV, with summary data in CD8 and CD4 T cells. One-way ANOVA test used to compare effect of EV from different donor groups ∗p < 0.05, ∗∗, p < 0.01, ∗∗∗p < 0.001. Wilcoxon signed rank test used to compare groups with the PBS control (i.e., to 1) §p < 0.05, §§p < 0.01, §§§p < 0.001, §§§§p < 0.0001.

T cell metabolism is tightly linked to effector capacity,^30^ thus we wondered whether EV may also alter T cell metabolism given the observed suppression of T cell effector cytokine production. Utilizing the same EV-T cell co-culture protocol, we first assessed T cell nutrient transporter abundance. We found both HD and Mild EV to be highly suppressive of glucose transporter 1 (GLUT-1) upregulation in CD8 and CD4 T cells, whereas Vac EV and Severe EV were less suppressive (Figures 3A–3C). HD and Mild EV also suppressed upregulation of the ferritin transporter CD71 in T cells, while Severe and Vac EV had weak or no suppressive impact on CD71 surface expression (Figures 3D and 3E).

To determine whether the suppression of GLUT-1 and CD71 expression was linked to an EV-induced alteration of T cell metabolism we used “Single Cell ENergetic metabolism by profiling Translation inHibition” (SCENITH), a recently published method allowing delineation of metabolic pathways used by single cells in mixed cell populations.^31^ SCENITH makes use of the understanding that cellular protein synthesis is energetically costly. Briefly, by measuring incorporation of puromycin into newly synthesized proteins, cellular dependence on a given metabolic pathway can be delineated by measuring changes in protein synthesis upon inhibition of that metabolic pathway (Figures S2A and S2B). We assessed metabolic dependence in T cells co-cultured with EV derived from vaccinated, mild or severe COVID-19 convalescent donors only, due to a limited availability of EV derived from unexposed healthy donor samples. We observed that in line with the finding that Mild EV had a generally suppressive effect, T cells treated with Mild EV, showed a reduction in general protein synthesis as determined by puromycin labeling (Figures 3F and S2C). T cells treated with EV derived from vaccinated donors also showed reduced protein synthesis, while protein synthesis in T cells treated with Severe EV was no different from PBS-treated control T cells (Figures 3F and S2C). To delineate whether glycolysis was implicated in fueling the observed protein synthesis, we used the glycolysis inhibitor 2-deoxy glucose (2DG) to block this pathway. In cells actively using glycolysis, blockade of this pathway is expected to reduce protein synthesis and thus puromycin labeling, while in cells fueling protein synthesis by other metabolic pathways, treatment with 2DG should have little impact on puromycin incorporation.^31^ Alterations in puromycin fluorescence intensity can then be used to calculate the cell’s usage or “dependence” on any given pathway using the formula outlined (Figure S2B and STAR methods section).

In our setting, T cells showed a trend toward an enhanced dependence on glycolysis to fuel protein synthesis following co-culture with EV derived from either vaccinated or severe COVID-19 convalescent donors (Figures 3F and S2D), whereas protein synthesis in T cells co-cultured with EV derived from mild convalescent donors showed a trend toward decreased dependence on glycolysis compared to the PBS control (Figures 3F and S2D). This is in line with our finding that Mild EV-treated T cells had reduced levels of glucose transporter GLUT-1 (Figures 3A–3C), suggesting reduced capacity to uptake glucose. To evaluate whether T cell mitochondrial function might also be influenced by EV, we made use of a well-established assay, measuring mitochondrial membrane polarization by utilizing a combination of MitoTracker-DeepRed, a dye that is polarization-dependent and stains functional mitochondria with polarized membranes, and MitoTracker-Green, which stains mitochondria independent of membrane polarization^32^^,^^33^; the Green(hi) DeepRed(lo) population is defined as containing dysfunctional non-polarized mitochondria. Mild EV increased mitochondrial dysfunction in both CD8 and CD4 T cells, while Vac and Severe EV did not (Figure 3G). Taken together our data indicate that exposure to EV can impact the metabolic signature of activated T cells, with EV derived from mild convalescent donors having an overall suppressive effect on metabolism as determined by reduced GLUT-1 and CD71 expression, as well as reduced glucose dependence and mitochondrial polarization.

### EV from mild COVID-19 convalescent donors can utilize ICAM-1 to interact with T cells

**Figure 4:**
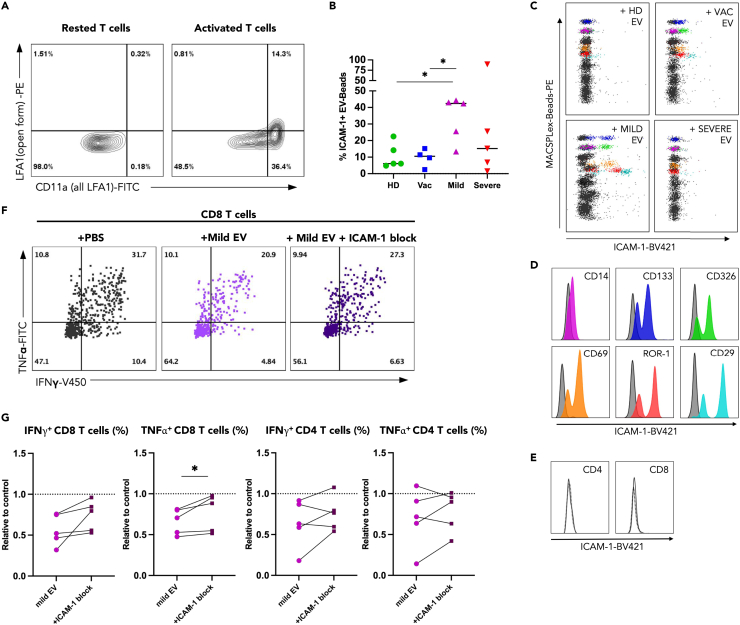
Impact of EV derived from mild COVID-19 convalescent donors on T cells can be partially abrogated through EV-ICAM-1 blockade (A) Flow cytometry staining of LFA-1 on rested and activated T cells, using an antibody against CD11a (the alpha chain of the LFA1 heterodimer) to stain all LFA1 and an antibody (clone m24) to specifically stain the open form of LFA1. (B) EV were analyzed by MACSPlex with additional surface ICAM-1 staining, percent of EV bound to MACSPlex beads that stained ICAM-1^+^ in each condition is shown. (C) Representative flow plots showing ICAM-1 staining on different EV populations by MACSPlex, 6 EV-bead populations that are ICAM-1^+^ in Mild covid convalescent EV are highlighted in all plots. (D) Colored histograms corresponding the highlighted EV populations in C that stained positive for ICAM, gray histograms are the blank control. (E) CD4 and CD8 EV-beads which did not stain positive for ICAM, blank control shown with dashed line. (F) CD8 and CD4 T cells were incubated with EV in co-culture assay as before, with or without prior EV-ICAM-1 blockade then IFNγ and TNFα were stained by flow cytometry, representative example shown (G) summary data of relative proportion of IFNγ^+^ and TNFα^+^ CD4 and CD8 T cells. All flow cytometry data are shown as values relative to the PBS control within the same experiment. One-way ANOVA test used to compare effect of EV from different donor groups ∗p < 0.05. Paired T test used to compare effect of ICAM-1 block within same donor cells ∗p < 0.05.

Our data indicated greater similarities in protein content between Mild EV and Vac EV (Figure 1), yet our functional data showed more pronounced suppression of T cell function was mediated by EV derived from mild COVID-19 convalescent donors. We therefore hypothesized the impact of EV on T cell function might be modulated at the level of T cell-EV recognition. Since EV and T cells in our system were derived from different non-HLA matched donors, interactions between EV-derived MHC and the T cell receptor, as previously described in the literature, were unlikely.^13^ An alternative documented route of interaction is mediated via binding of intercellular adhesion molecule 1 (ICAM-1/CD54) on EV to lymphocyte function-associated antigen-1 (LFA-1) on activated T cells.^34^ The open form of the LFA-1 heterodimer, conferring high affinity for ICAM-1, was induced upon T cell activation (Figure 4A), thus we probed whether the ICAM-1/LFA1 axis may be a route of EV-T cell interaction in our setting. To assess the presence of ICAM-1 on plasma-derived EV, we customized the commercial MACSPlex by addition of an ICAM-1-targeting antibody (Figures 4B–4D). ICAM-1 could be detected on EV but tended to be increased in EV from donors post-mild infection (Figures 4B and 4C), which could lead to increased ability of this group to communicate with T cells via this route. This finding is in line with the increased impact of Mild EV on T cell function observed (Figures 2 and 3); it is worth noting that the outlier present in the Severe EV group with high ICAM-1 expression *was* able to suppress CD4 and CD8 T cell cytokine production by ∼40% (data not shown). Interestingly, ICAM-1 was present only on EV of specific cellular origin, namely CD14^+^, CD133^+^, CD326^+^, CD69^+^, ROR-1^+^, and CD29^+^ EV (Figure 4D), suggesting a leading role of these producer cells in shaping the convalescent T cell response, post-mild COVID-19 disease. In contrast, ICAM-1 was not present on CD4^+^ or CD8^+^ EV (Figure 4E), suggesting T cell-derived EV likely does not play a significant role in our system.

To establish whether ICAM-1 expression was required for the interaction of Mild EV with activated T cells, we treated Mild EV with an anti-ICAM-1 blocking antibody prior to co-culture. Given the impact of Mild EV on T cell IFNγ and TNFα production (Figure 2), these cytokines were used as the assay readout. Indeed, blockade of EV-expressed ICAM-1 partially rescued the proportion of cytokine^+^ T cells (Figures 4F and 4G), with the most pronounced effect observed in CD8 T cells (Figure 4G). Our data suggests that the ICAM-1/LFA-1 axis is one of the routes by which EV deliver their immune-suppressive signals to T cells and sheds light on the ICAM-1^+^ EV populations involved in this process post mild COVID-19 disease.

## Discussion

We show here that at several weeks following immune challenge by mild and severe COVID-19 disease or mRNA vaccination, circulating EV remain compositionally and functionally distinct from those in healthy donors. We performed extensive surface marker profiling on circulatory EV, as the expression of EV surface markers conveys valuable information about the type of cells releasing EV at the point of plasma isolation. We show that different cell types are actively releasing EV during COVID-19 convalescence and following vaccination, and that recovery from mild versus severe disease involves distinct EV types. We were particularly interested to see that in most cases CD4 T cells do not release detectable EV during homeostasis, yet following immune challenge by infection or vaccination nearly all donors had detectable CD4^+^ EV. Conversely, CD8^+^ EV were abundant in healthy donors and present in high numbers following severe COVID-19 disease. These data would suggest unique roles for CD4 and CD8 T cell communication in resolving an immune response to infection and vaccination. T cells release more EV upon TCR stimulation and proliferation^35^ therefore the increased presence of CD8^+^ EV in severe COVID-19 convalescent donors may be attributed to recovery of T cell numbers following attrition that has been reported during severe disease.^36^^,^^37^ Additionally, there was a high proportion of platelet-derived EV in severe COVID-19 convalescent donors. Platelet EV have been shown to enhance thrombosis^38^ which could suggest an involvement of EV in the increased incidence of thrombotic events following severe COVID-19.^39^ Interestingly, in mild COVID-19 convalescent donors there was a high proportion of antigen presenting cell-derived and endothelial cell-derived EV, both of which have been implicated in immune suppression.^40^ Interrogation of intra-vesicle proteins further conveyed that EV from mild and severe COVID-19 convalescent donors were highly distinct. Clustering based on both EV surface and intra-vesicle protein abundances allowed partial segregation of mild and severe COVID-19 convalescent donors further demonstrating distinct EV characteristics.

To establish the role of EV in immune-regulation post COVID-19, we interrogated the impact of convalescent plasma EV on T cell function and metabolism. In line with their different cellular origins, EV derived from healthy, vaccinated, mild, and severe COVID-19 convalescent donors all had distinct functional impacts on CD8 and CD4 T cells, suggesting different roles for EV in these settings. Interestingly, we found EV during health to induce metabolic down-modulation in T cells compared to conventional culture without EV, reducing expression of the nutrient transporters CD71 and GLUT-1. This suggests a primary role of EV in immune-regulation and maintenance during homeostasis. Intriguingly, EV following vaccination were functionally distinct from those during health. Post-vaccination EV induced specific activation of CD4 T cells as measured by increased CD69 expression and induced higher CD71 and GLUT-1 expression compared to healthy donor EV. This, combined with the high proportion of CD4^+^ EV, might suggest a potential role for EV in facilitating the T-helper response following vaccination. The most striking impact on T cells was mediated by EV isolated from mild COVID-19 convalescent donors; these EV significantly suppressed IFNγ and TNFα effector cytokine production in both CD4 and CD8 T cells as well as inducing metabolic modulation; specifically, nutrient transporter downregulation and mitochondrial dysfunction. The suppressive impact of mild COVID-19 convalescent EV on T cell function might be supported by their packaging of immune-suppressive proteins as determined by intra-vesicle proteomics. Speculatively, this heightened immune-suppressive function might be beneficial by helping to dampen the T cell response following infection and bring a return to immune-homeostasis. It is possible that the regulatory potential of EV in donors recovered from mild infection already played a role during the acute phase, facilitating the mild outcome. This possibility remains to be investigated. The functional impact of EV following a severe infection is highly different from the mild COVID-19 setting. EV from severe COVID-19 convalescent donors were less able to suppress cytokine production by healthy T cells and had a weaker impact on T cell metabolism. This lack of immune-regulatory function of Severe EV may play a role in the dysregulated state of the immune system during and after severe COVID-19 disease and might be contributing to the long-covid symptoms that are experienced by a greater proportion of convalescent donors recovering from severe disease.^41^ It will be interesting to interrogate these findings in more detail in the future, including the impact of EV on other immune cell populations.

The differential functional impact of EV following mild versus severe infection is in line with their distinct surface and intra-EV protein contents. However, we were interested to find that EV following vaccination were not functionally akin to EV following mild COVID-19 disease considering they showed high similarity in protein cargo. We therefore propose that determining the routes by which EV and immune cells interact in a given disease will be crucial for predicting EV-mediated impact and designing future therapies. It has been shown previously that activated T cells can recruit EV via LFA-1/ICAM-1 interaction and blockade of this interaction reduces EV uptake.^34^ In our cohort, we detected the highest presence of ICAM-1 on EV derived from donors recovered from mild COVID-19 disease. Indeed, by blockade of ICAM-1 on Mild EV we could partially revert the impact of EV on activated T cells. Of note, we show that only distinct types of EV display surface ICAM-1, with the highest expression on EV with CD326^+^ (epithelial cell adhesion molecule Epcam), ROR-1^+^ (cell surface tyrosine kinase) and CD29^+^ (very late antigen 4 (VLA-4)/Intergrin beta 1), indicating that cell populations positive for these markers can engage in the regulation of activated T cells via this route. This finding sheds light on the wider immune regulatory network at play and warrants further investigation.

Taken together, our data demonstrate that infection with SARS-CoV-2 has long-term impacts on the profile of circulating EV, with EV surface and intra-vesicle protein content linked to initial disease severity. We provide evidence that EV can modulate T cell responses several weeks post COVID-19 disease, impacting T cell activation, metabolic profile, and effector functions. Specifically following mild infection, EV are suppressive to T cell function. A lack of immune-suppressive EV in circulation following severe infection may therefore contribute to the prolonged T cell activation that has been reported. Future work to establish the characteristics of EV following other viral infections, the longevity of their immune-modulatory effects and the specific cell types that release them, will help to elucidate the role of cell-cell communication in the long-term outcome of infection.

### Limitations of the study

The limited number of donors in each group means that this is an observational study. The pre-COVID-19 pandemic unexposed healthy donor controls were selected to match the study groups regarding age and sex as closely as possible, however, the average age of the vaccinated donors was lower than the healthy cohort. The severe COVID-19 convalescent cohort contained some older individuals due to the nature of increased susceptibility to severe disease with age; as such age may be a factor in some of the differences observed. We isolated EV by CD63^+^ immune-beads and while we found this method to be optimal for EV purification, it does mean that we are not investigating the total EV pool. We have here assessed the protein content of EV, but not other cargo such as miRNA. In future work this would be relevant to interrogate, given the immunomodulatory functions of many miRNAs known to be packaged among the total cargo of EV.

## STAR★Methods

### Key resources table

**Table:** 

REAGENT or RESOURCE	SOURCE	IDENTIFIER
**Antibodies**		

Mouse monoclonal APC-Cy7 conjugated anti-CD3	BioLegend	CAT#344818; RRID:AB_10645474
Mouse monoclonal PE-CY7 conjugated anti-CD4	BioLegend	CAT#317414; RRID:AB_571959
Mouse monoclonal Alexa Fluor700 conjugated anti-CD8	Thermo Fisher Scientific	CAT#56-0086-82; RRID:AB_657756
Mouse monoclonal Brilliant Violet 605 conjugated anti-CD69	BioLegend	CAT#310938; RRID:AB_2562307
Mouse monoclonal PE conjugated anti-PD1	BioLegend	CAT#329906; RRID:AB_940483
Mouse monoclonal Per-CP-Cy7 conjugated anti-CD71	BioLegend	CAT#334114; RRID:AB_2563175
Mouse monoclonal FITC conjugated anti-TNFa	BioLegend	CAT#502906; RRID:AB_315258
Mouse monoclonal Violet-450 conjugated anti-IFNg	Becton Dickinson	CAT#560371; RRID:AB_1645594
Rabbit monoclonal APC conjugated anti-Glut1	Abcam	CAT#Ab195020; RRID:AB_2783877
Ultra-LEAF purified mouse monoclonal anti-CD3, clone OKT3	BioLegend	CAT#317326; RRID:AB_11150592
Mouse monoclonal anti-CD28, clone CD28.2	BioLegend	CAT#302902; RRID:AB_314304
Rabbit monoclonal Alexa Fluor-488 conjugated anti-puromycin	Merck	CAT#MABE343-AF488; RRID:AB_2736875
Mouse monoclonal PE conjugated anti-LFA-1, clone m24	BioLegend	CAT#363405; RRID:AB_2721626
Mouse monoclonal FITC conjugated anti-CD11a	BioLegend	CAT#350604; RRID:AB_10662904
Ultra-LEAF purified mouse monoclonal anti-ICAM, clone HCD54	BioLegend	CAT#322721; RRID:AB_2832632

**Chemicals, peptides, and recombinant proteins**		

Brefeldin-A	Biolegend	420601
MitoTracker Deep Red FM	ThermoFisher	M22426
MitoTracker Green FM	ThermoFisher	M7514
2-Deoxy-D-Glucose (99% purity)	Merck	D6134
Oligomycin-A	Selleck Chem	No.S1478
Puromycin	Merck	P7255

**Critical commercial assays**		

Exosome Isolation Kit CD63	Miltenyi Biotec	130-110-918
Pierce BCA Protein Assay Kit	ThermoFisher Scientific	23227
MACSPlex EV kit, human	Miltenyi Biotec	130-108-813
Pan T cell Isolation Kit	Miltenyi Biotec	130-096-535
FOXP3 Fix/Perm Kit	ThermoFisher	00-5523-00
Cytofix/Cytoperm	BD	51-2090KZ

**Deposited data**		

Proteomics data	This paper	PXD043058

**Software and algorithms**		

FlowJo v10	FlowJo, LLC	https://www.flowjo.com/
ClustVis Software	Metsalu et al.^42^	https://biit.cs.ut.ee/clustvis/
R v4.1.1 with limma and q-value packages	The R Foundation	https://www.r-project.org/
Spectronaut v15	Biognosys	https://biognosys.com/software/spectronaut/
DAVID analysis tool	Dennis Jr. et al.^43^	https://david.ncifcrf.gov/

**Other**		

S-trap micro columns	Profiti	C02-micro-10

### Resource availability

#### Lead contact

Further information and requests for resources and reagents should be directed to and will be fulfilled by the lead contact, Anna Schurich (anna.schurich@kcl.ac.uk).

#### Materials availability

This study did not generate new unique reagents.

### Experimental model and study participant details

#### Ethics statement

Blood samples from healthy volunteers were obtained under The Guy’s and St Thomas’ License (license number 12121). All subjects gave their informed written consent for inclusion before they participated in the study. The study was conducted in accordance with the Declaration of Helsinki, and the protocol for healthy volunteer recruitment and sampling was approved by the committee of the Infectious Diseases Biobank of King’s College London with reference number AS1-280119. The approval was granted under the terms of the Infectious Disease Biobank’s ethics permission (REC ref. 19/SC/0232) granted by the South-Central Hampshire B Research Ethics Committee. Approval was granted for the recruitment of COVID-19 convalescent donors under the terms of a clinical research study to investigate immunological lung disease (REC ref. 14/LO/1699). All storage of samples obtained complied with the requirement of the Data Protection Act 1998 and the Human Tissue Act 2004, issued by the UK parliament.

#### Patient recruitment and sampling

Plasma samples were obtained from healthy donors, vaccinated donors and donors recovered from mild or severe COVID-19, 2-10 weeks after symptoms began or after a positive test and stored at -80°C prior to use. Peripheral blood to assess T cell function was obtained from healthy donors and isolated PBMCs were stored in liquid nitrogen prior to use.

### Method details

#### Isolation of EV

Plasma was collected from whole blood by density gradient centrifugation and stored at -80°C prior to use. Cryopreserved plasma samples were thawed and cleared of cell and larger vesicle debris by serial centrifugation at 2000 × g for 30 mins then 10000 × g for 45 mins. EV were isolated from 2 mL cleared plasma using an immuno-bead isolation kit (Exosome isolation kit, CD63, Miltenyi Biotec), as per manufacturer’s instructions. Following isolation, protein content was determined by BCA assay (Pierce BCA Protein Assay Kit, ThermoFisher Scientific) as per instructions.

#### Multiplex EV assay and flow cytometry analysis

Samples were analysed by a multiplex bead-based flow cytometry assay (MACSPlex EV kit, human, Miltenyi Biotec).^4^ For each sample, 12μg EV were incubated in 120 μL MACSPlex buffer with 15 μL capture beads overnight in an orbital shaker at 450rpm. After 16-20 hours samples were washed with MACSPlex buffer then incubated for 1 hour with 10 μL detection cocktail (APC-CD81, APC-CD9) then washed again. Samples were acquired for 70 seconds on a BD LSR Fortessa 3 (Becton Dickinson) flow cytometer. Analysis was performed with FlowJo software v10.

#### EV and T cell co-culture assay

Peripheral blood mononuclear cells (PBMCs) were isolated from whole blood by density gradient centrifugation (Lymphoprep, STEMCELL Technologies, Cambridge, UK). Isolated PBMCs were preserved in FCS +10% DMSO and stored in liquid nitrogen prior to use. Cryopreserved PBMCs were thawed rapidly at 37°C and washed with 1x PBS, T cells were isolated by pan T cell isolation kit (Miltenyi Biotec) as per the protocol. T cells were resuspended in RPMI 1640 medium supplemented with 10mM glucose, 0.1mM non-essential amino acids, 10mM HEPES buffer, 1mM sodium-pyruvate, 50 IU/mL of streptomycin and 50 IU/mL penicillin (all Sigma-Aldrich) and 10% FCS. T cells were kept in a 96 well plate at 200,000 cells/ well in an incubator at 37°C, 5% CO_2_. On Day 1, T cells were stimulated with plate-bound anti-CD3 (OKT3 clone, 1 μg/mL) and anti-CD28 (CD28.2 clone, 0.5 μg/mL) (BioLegend) overnight with 20 IU/mL of interleukin (IL)-2. On day 2, T cells were removed from stimulation to rest. On day 5, cells were replenished with fresh media and 20 IU/mL IL-2. On day 5 and day 6, 5μg EV in 25 ul 1X PBS were added per well. On day 7 cells were restimulated with plate-bound anti-CD3 and anti-CD28 (BioLegend) and 1 μg/mL Brefeldin A (BFA; Biolegend) was added per well. Cells were analysed on day 8.

#### T cell phenotyping and cytokine analysis

Cells were washed with 1xPBS then stained for surface markers with fluorophore-conjugated antibodies in 1x PBS for 30 min at 4°C protected from light. The following antibodies were used for staining: CD3-APC-Cy7, CD4-PE-Cy7, CD8-Alexa-Fluor-700, CD69-Brilliant Violet-605, PD-1-PE, CD71-Per-CP-Cy7, LFA-1-PE, CD11a-FITC (all BioLegend). Cells were stained concurrently with 1 μL/mL Live/Dead dye (ThermoFisher Scientific). Cells were washed with 1x PBS then fixed with fix/perm buffer set (BD Biosciences) for 20 min at 4°C protected from light. Cells were stained for intracellular markers using fluorophore conjugated antibodies in permeabilization buffer (1% FCS, 0.1% saponin in 1x PBS). The following intracellular antibodies were used: GLUT-1-APC (Abcam), TNF-α-FITC and IFN-γ-V450 (Becton Dickinson). Samples were run on a BD Fortesssa. FlowJow v10 software was used for the analysis.

#### T cell mitochondria staining

Cells were stained with 50 nm Mitotracker Deep Red FM and 5nm Mitotracker Green FM (thermofisher) in RPMI for 20 minutes at 37°C, 5% CO_2_. Cells were washed with ice-cold PBS and stained for surface markers prior to flow cytometry analysis.

#### Metabolic flux analysis (SCENITH)

Method adapted from work published by Arguello et al.^32^ T cells (1 million/mL) were incubated for 45 min with puromycin (20 μg/mL) and either 1xPBS, 2-deoxy-D-glucose (2DG, 100mM) or a combination of 2DG and Oligomycin-A (1μM). T cells were washed in ice-cold PBS then stained for surface markers as previously described. Puromycin was stained intracellularly using the FOXP3 fix/perm kit (Thermofisher) using AF488-puromycin antibody (Merck). The following calculation was used to determine glucose dependence, as per the published protocol:^32^

$$Glucose\;Dependence\;\left(\%\right)=\left(\frac{Puro\;{GMFI}_{PBS\;control}-Puro\;{GMFI}_{2DG}}{{Puro\;GMFI}_{PBS\;control}-{Puro\;GMFI}_{2DG+Oligomycin}}\right)\times 100$$

#### EV ICAM-1-blockade assay

Isolated EV were incubated with an anti-ICAM-1 blocking antibody (clone: HCD54) for 1 hour, EV-beads were washed free of any unbound anti-ICAM-1 by running through a magnetic MACS column and washing with PBS. EV-anti-ICAM-1 were then added to the EV coculture assay as described, alongside EV alone.

#### Proteomics

EV samples were processed for proteomic analysis using the S-trap method. Briefly, 2 μg of isolated EV in PBS were lysed in an equal volume of 2x concentrated S-trap lysis buffer to a final concentration of 5% SDS, 10 mM TCEP and 50 mM TEAB. Samples were shaken at 1000 rpm for 30 minutes at room temperature, then boiled at 95°C for 5 mins with shaking at 500 rpm, then at 1000 rpm for 5 mins at room temperature. Samples were sonicated briefly, then incubated with benzonase for 15 mins at 37°C to shear any DNA. To alkylate, freshly made iodoacetamide was added to a final concentration of 20mM and samples incubated for 1hr in the dark at room temperature. Samples were then processed using the S-trap micro columns (Profiti) according to the manufacturer’s instructions. For protein digestion, samples were incubated with 1 μg trypsin for 2 hrs at 47°C. Following digestion, peptides were eluted from the S-trap column and dried down in a SpeediVac.

Mass spectrometry analysis was performed by the FingerPrints Proteomics Facility, University of Dundee. Peptides were resuspended in 18 μL 1% formic acid and 15 μL injected onto a nanoscale C18 reverse-phase chromatography column coupled to an UltiMate 3000 RSLC nano HPLC system (Thermo Fisher) and an Orbitrap Exploris 480 Mass Spectrometer (Thermo Fisher). Loaded peptides were eluted from the resolving C18 column (75 μm × 50 cm, PepMap RSLC C18 column, 2 μm, 100 Å) with a flow of 300 nL/min and a gradient of 3% buffer B to 6% buffer B in 5 mins, then 6% buffer B to 35% buffer B in 115 mins, then 80% buffer B in 7 mins, where buffer A is 0.1% formic acid and buffer B is 0.1% formic acid/80% acetonitrile. Data was acquired using an easy spray source operated in positive mode, and the mass spectrometer was operated in DIA mode, with a scan cycle consisting of a full MS scan (m/z range 350 – 1650). Data for MS and MS/MS scans were acquired in profile mode.

Raw files were searched using Spectronaut version 15 using a library generated from the EV samples and a Swiss-Prot human database downloaded in July 2020. An inverse decoy library was used, and precursor and protein Q-value cut-off of 0.01 (0.05 for proteins per run). Quantification was done using the Quant 2.0 LFQ method and with the major and minor group quantity set as sum peptide quantity and sum precursor quantity respectively. Cross-run normalization was deselected. The resulting intensity data was further analysed in R (version 4.1.1) with data normalised with the cyclic loess method using the limma package. Differential expression analysis was performed using the limma and q-value packages. Significantly regulated proteins were defined as those with a q-value < 0.1. For pathway enrichment analysis, proteins were submitted to the DAVID analysis tool with the whole human proteome as background, with enriched pathways defined as those with a q-value < 0.05.

### Quantification and statistical analysis

Statistical analysis of proteomics data is described in the proteomics STAR methods section. Prism 9 (GraphPad) was used for all other analyses. Statistical significance was determined using Kruskal Wallis multiple comparisons test to compare impact of different EV groups with each other, or a Wilcoxon Signed Rank test was used to compare each group with the PBS control set to 1. P < 0.05 was considered statistically significant.

## Data Availability

• This paper does not report original code. • The mass spectrometry proteomics data have been made public and deposited to the ProteomeXchange Consortium via the PRIDE^44^ partner repository with the dataset identifier PXD043058. • Other data available upon request from the lead contact, Anna Schurich.
