# Supplementary material for: Extracellular vesicles in COVID-19 convalescence can regulate T cell metabolism and function
Source: iScience. 2023 Jul 4;26(8):107280. doi: 10.1016/j.isci.2023.107280 (PMC10371842; doi:10.1016/j.isci.2023.107280)
Supplement: Document S1. Figures S1 and S2 [file mmc1.pdf]

iScience, Volume *26*

Supplemental information

Extracellular vesicles in COVID-19 convalescence can regulate T cell metabolism and function

Molly S. George, Jenifer Sanchez, Christina Rollings, David Fear, Peter Irving, Linda V. Sinclair, and Anna Schurich

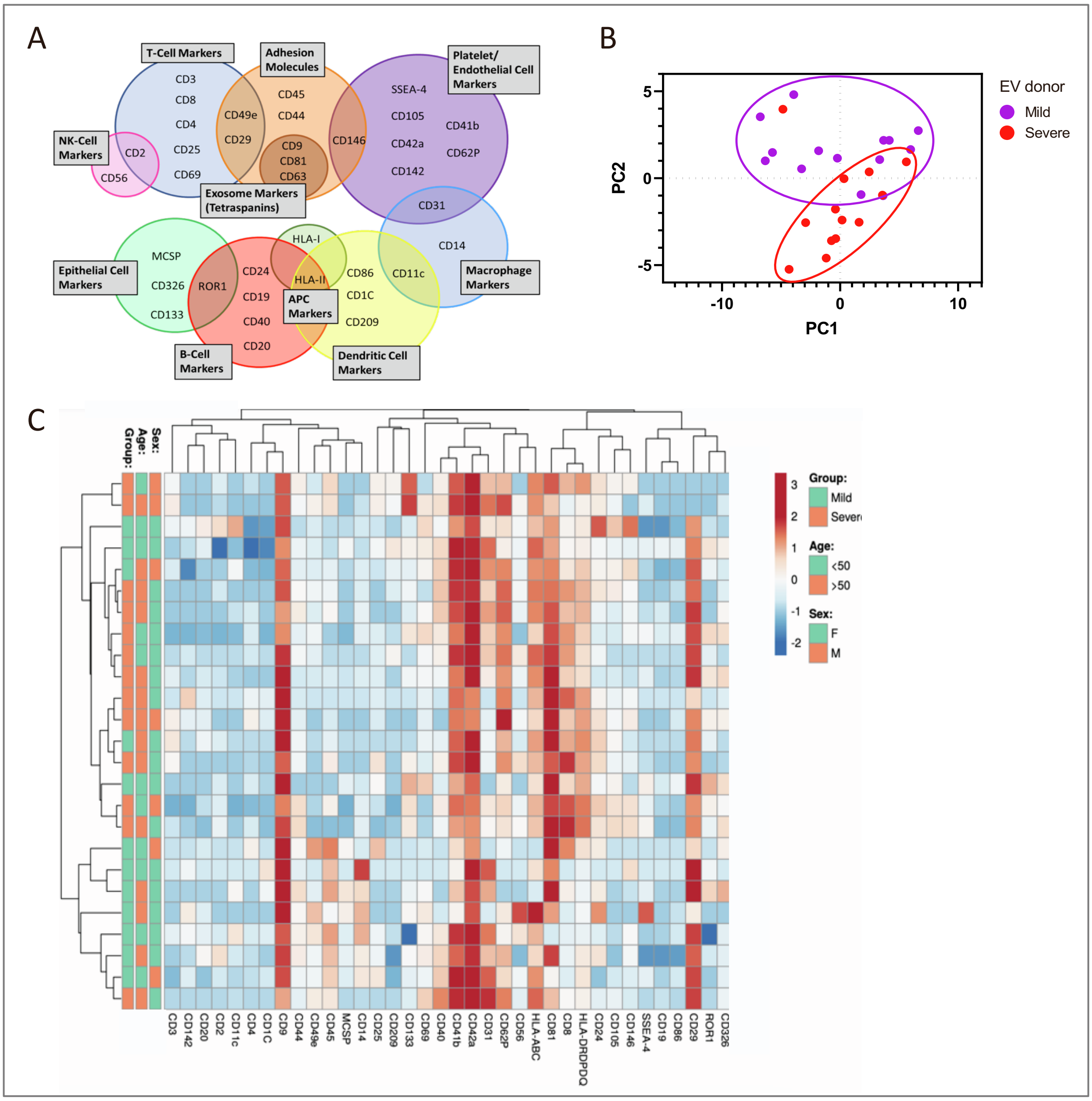

Supplementary Figure 1 (related to main text Figure 1): Plasma-derived EVs carry distinct surface and intra-vesicle proteins in COVID-19 convalescent donors. EV surface markers were analysed by a multiplexed flow cytometry method (MACSPlex). **(A)** Schematic representation of the surface markers detectable by MACSPlex assay. **(B)** PCA analysis of GMFI for 36 markers detected by MACSPlex in Mild vs Severe donor EV. **(C)** Unsupervised clustering of data as in B created using ClustVis software, original values are ln(x + 1)-transformed. Rows are centered; unit variance scaling is applied to rows. Rows are clustered using correlation distance and Ward linkage. Columns are clustered using correlation distance and average linkage. Data is annotated with EV donor group (Mild or Severe COVID-19 convalescent), age of donor and sex of donor.

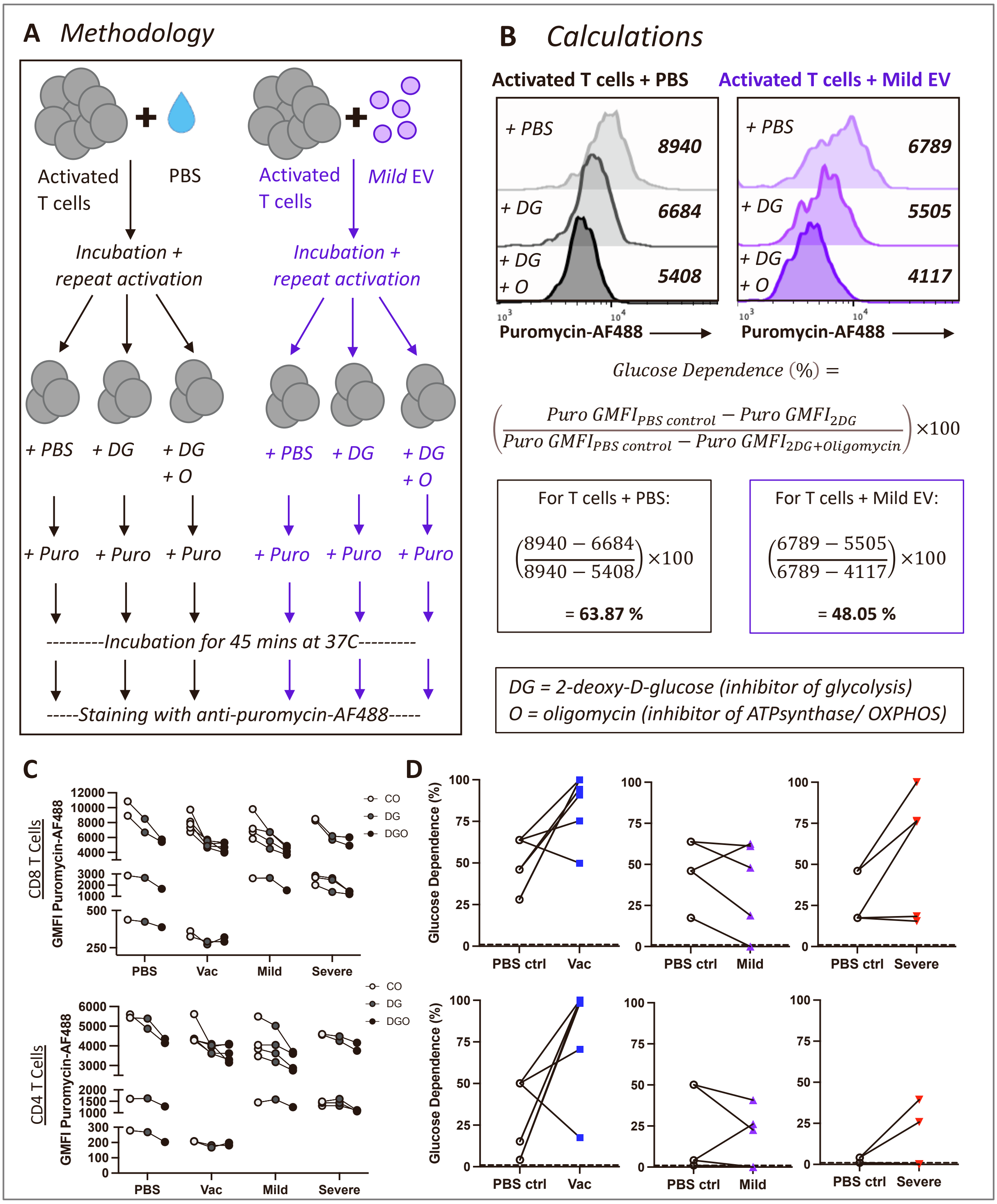
A Methodology
Activated T cells
PBS
Activated T cells
Mild EV
Incubation + repeat activation
Incubation + repeat activation
+ PBS
+ DG
+ DG + O
+ PBS
+ DG
+ DG + O
+ Puro
+ Puro
+ Puro
+ Puro
+ Puro
+ Puro
---------Incubation for 45 mins at 37C---------
-----Staining with anti-puromycin-AF488-----
B Calculations
Activated T cells + PBS
Activated T cells + Mild EV
+ PBS 8940
+ DG 6684
+ DG + O 5408
+ PBS 6789
+ DG 5505
+ DG + O 4117
Puromycin-AF488
Puromycin-AF488
Glucose Dependence (%) =
$\left(\frac{Puro\ GMFI_{PBS\ control} - Puro\ GMFI_{2DG}}{Puro\ GMFI_{PBS\ control} - Puro\ GMFI_{2DG+Oligomycin}}\right) \times 100$
For T cells + PBS:
$\left(\frac{8940 - 6684}{8940 - 5408}\right) \times 100$
= 63.87 %
For T cells + Mild EV:
$\left(\frac{6789 - 5505}{6789 - 4117}\right) \times 100$
= 48.05 %
DG = 2-deoxy-D-glucose (inhibitor of glycolysis)
O = oligomycin (inhibitor of ATPsynthase/ OXPHOS)
C
CD8 T Cells
GMFI Puromycin-AF488
CO
DG
DGO
PBS
Vac
Mild
Severe
CD4 T Cells
GMFI Puromycin-AF488
D
Glucose Dependence (%)
PBS ctrl
Vac
Mild
Severe

Supplementary Figure 2 (related to main text Figure 3F): EV derived from COVID-19 convalescent donors differentially impact the metabolism of healthy CD8 and CD4 T cells. T cells were stimulated and treated with EV from the different donor groups or with PBS as a control. Following co-culture, functional metabolism was assessed. **(A)** Methodology for assessing functional metabolism; cells are divided into three wells and incubated with either PBS, DG (2-deoxy-d-glucose, glycolysis inhibitor) or a combination of DG and O (Oligomycin, ATP synthase inhibitor). All cells are then incubated with puromycin for 45 minutes and then stained with anti-puromycin. **(B)** An outline of the calculations required to determine glucose dependence. Calculations are performed using the GMFI values for puromycin-AF488 in each condition. Representative examples of flow cytometry histograms for control T cells and T cells incubated with Mild EV are shown. **(C)** Puromycin-AF488 GMFI values for each condition (PBS, DG, DG+O) for each set of experiments conducted with T cells alone (+PBS) and T cells incubated with the different EV groups (Vac, Mild, Severe). **(D)** Glucose dependence of CD8 (top) and CD4 (bottom) T cells following incubation with EV compared to PBS control. Values in C and D are the raw values used to generate the normalised data shown in main text figure 3F.
